# Supplementary material for: GDC 2: Compression of large collections of genomes
Source: Sci Rep. 2015 Jun 25;5:11565. doi: 10.1038/srep11565 (PMC4479802; doi:10.1038/srep11565)
Supplement: Supplementary Information [file srep11565-s1.pdf]

Supplementary information
GDC 2: Compression of large collections of genomes

Sebastian Deorowicz[1,*], Agnieszka Danek[1], and Marcin Niemiec[2]

[1]Institute of Informatics, Silesian University of Technology, Akademicka 16, 44-100 Gliwice, Poland
[2]Nubitech, 40-684 Katowice, Poland
[*]sebastian.deorowicz@polsl.pl

1 Detailed data description

1.1 The *H.sapiens* dataset

The *H.sapiens* dataset contains 2184 strains from the Phase 1 of the 1000 Genomes Project. They are compressed against single reference sequence.

1.1.1 Reference sequence

The Genome Reference Consortium Human Build 37 was used as a reference sequence. Its complete sequence can be found at the NCBI's anonymous FTP server (`ftp://ftp-trace.ncbi.nih.gov//1000genomes/ftp/technical/reference/ /human_g1k_v37.fasta.gz`).

The reference sequences of assembled chromosomes can be found at the GenBank and downloaded from the NCBI's anonymous FTP server:
`ftp://ftp.ncbi.nlm.nih.gov/genbank/genomes/Eukaryotes/vertebrates_mammals/ Homo_sapiens/GRCh37/Primary_Assembly/assembled_chromosomes/FASTA/`
The complete list of compressed reference sequences:
`chr1.fa.gz`, `chr2.fa.gz`, `chr3.fa.gz`, `chr4.fa.gz`, `chr5.fa.gz`, `chr6.fa.gz`, `chr7.fa.gz`, `chr8.fa.gz`, `chr9.fa.gz`, `chr10.fa.gz`, `chr11.fa.gz`, `chr12.fa.gz`, `chr13.fa.gz`, `chr14.fa.gz`, `chr15.fa.gz`, `chr16.fa.gz`, `chr17.fa.gz`, `chr18.fa.gz`, `chr19.fa.gz`, `chr20.fa.gz`, `chr21.fa.gz`, `chr22.fa.gz`, `chrX.fa.gz`, `chrY.fa.gz`.

1.1.2 1000 Genomes Project

The 1000 Genomes Project (1000GP), "A Deep Catalog of Human Genetic Variation", describes the whole-genome sequence variation in human individuals. In out research we used data from Phase 1 of the 1000GP containing information about genomes of 1092 people, 525 males and 567 females. As the provided genotypes are phased, there are 2184 sequences in total (1092 diploid genomes).

The FASTA sequences were retrieved based on the VCF (Variant Call Format) files, which can be found at the 1000GP anonymous FTP servers. All used VCF files can be downloaded from the directory containing the final variant calls for the phase 1 datasets. It can be either the EBI or NCBI FTP site:

ftp://ftp.1000genomes.ebi.ac.uk/vol1/ftp/phase1/analysis_results/integrated_call_sets/

ftp://ftp.ncbi.nih.gov//1000genomes/ftp/phase1/analysis_results/integrated_call_sets/

The complete list of compressed VCF files used:

```
ALL.chr1.integrated_phase1_v3.20101123.snps_indels_svs.genotypes.vcf.gz
ALL.chr2.integrated_phase1_v3.20101123.snps_indels_svs.genotypes.vcf.gz
ALL.chr3.integrated_phase1_v3.20101123.snps_indels_svs.genotypes.vcf.gz
ALL.chr4.integrated_phase1_v3.20101123.snps_indels_svs.genotypes.vcf.gz
ALL.chr5.integrated_phase1_v3.20101123.snps_indels_svs.genotypes.vcf.gz
ALL.chr6.integrated_phase1_v3.20101123.snps_indels_svs.genotypes.vcf.gz
ALL.chr7.integrated_phase1_v3.20101123.snps_indels_svs.genotypes.vcf.gz
ALL.chr8.integrated_phase1_v3.20101123.snps_indels_svs.genotypes.vcf.gz
ALL.chr9.integrated_phase1_v3.20101123.snps_indels_svs.genotypes.vcf.gz
ALL.chr10.integrated_phase1_v3.20101123.snps_indels_svs.genotypes.vcf.gz
ALL.chr11.integrated_phase1_v3.20101123.snps_indels_svs.genotypes.vcf.gz
ALL.chr12.integrated_phase1_v3.20101123.snps_indels_svs.genotypes.vcf.gz
ALL.chr13.integrated_phase1_v3.20101123.snps_indels_svs.genotypes.vcf.gz
ALL.chr14.integrated_phase1_v3.20101123.snps_indels_svs.genotypes.vcf.gz
ALL.chr15.integrated_phase1_v3.20101123.snps_indels_svs.genotypes.vcf.gz
ALL.chr16.integrated_phase1_v3.20101123.snps_indels_svs.genotypes.vcf.gz
ALL.chr17.integrated_phase1_v3.20101123.snps_indels_svs.genotypes.vcf.gz
ALL.chr18.integrated_phase1_v3.20101123.snps_indels_svs.genotypes.vcf.gz
ALL.chr19.integrated_phase1_v3.20101123.snps_indels_svs.genotypes.vcf.gz
ALL.chr20.integrated_phase1_v3.20101123.snps_indels_svs.genotypes.vcf.gz
ALL.chr21.integrated_phase1_v3.20101123.snps_indels_svs.genotypes.vcf.gz
ALL.chr22.integrated_phase1_v3.20101123.snps_indels_svs.genotypes.vcf.gz
ALL.chrX.integrated_phase1_v3.20101123.snps_indels_svs.genotypes.vcf.gz
ALL.chrY.phase1_samtools_si.20101123.snps.low_coverage.genotypes.vcf.gz
```

For all individuals, variant calls for chromosomes 1–22 (autosomes) ale diploid and genotypes are phased. Therefore, the information about variants found on both chromosomes of chromosome pairs 1–22 is available for all individuals and it is possible to obtain 2184 sequences (2 for each individual). The data contain also information about pairs of chromosomes X for females (denoted by X-fem in the presented results). The situation is, however, more complex for male individuals due to two pseudoautosomal regions (PAR1 and PAR2) that are common between X and Y chromosomes. These pseudoautosomal regions are treated as autosomes in the description of the results of the 1000GP. Precisely, the diploid genotype calls for variants in two pseudoautosomal regions of X and Y chromosomes are stored in the available VCF file for X chromosome, while haploid genotype calls for non-pseudoautosomal regions (nonPAR, between PAR1 and PAR2) of X and Y chromosomes are stored in the corresponding VCF files (nonPAR of X in the VCF file for X chromosome, nonPAR of Y in the VCF file for Y chromosome).

Therefore, processing of X and Y chromosomes is divided into processing of five separate groups (which are treated as separate chromosomes):

- X-fem—whole chromosome X of females, with diploid genotype calls (2 sequences per individual),

- X-mal—nonPAR region of chromosome X of males, with haploid genotype calls (1 sequence per individual),
- Y-mal—nonPAR region of chromosome Y of males, with haploid genotype calls (1 sequence per individual),
- XY-mal1— PAR1 region of X and Y chromosomes of males, with diploid genotype calls (2 sequences per individual),
- XY-mal2— PAR2 region of X and Y chromosomes of males, with diploid genotype calls (2 sequences per individual).

Note that the above split is only according to the phasing status of the PAR regions in the 1000GP data. We had to use such split as we needed genome sequences to examine the existing compressors.

The scripts to generate FASTA sequences using VCF files from the Phase 1 of the 1000GP are available together with the distribution of GDC 2 (see Section 2.2).

1.2 The *A.thaliana* dataset

The *A.thaliana* dataset contains 775 strains from the 1001 Genomes Project. They are compressed against single reference sequence.

1.2.1 Reference sequence

The *A.thaliana* genome TAIR10 assembly was used as the reference sequence. It can be accessed on the anonymous FTP server:
`ftp://ftp.arabidopsis.org//Sequences/whole_chromosomes/`.
The complete list of compressed reference sequences of chromosomes:
`TAIR10_chr1.fas`, `TAIR10_chr2.fas`, `TAIR10_chr3.fas`, `TAIR10_chr4.fas`, `TAIR10_chr5.fas`, `TAIR10_chrC.fas`, `TAIR10_chrM.fas`.

1.2.2 1001 Genomes Project

The 1001 Genomes Project (1001GP),"A Catalog of *A.thaliana* Genetic Variation", contains the whole-genome sequence variation in strains accessions of the plant *A.thaliana*.

The 775 FASTA sequences were retrieved based on the available information about variants found in related individuals. Described variants were found on the 5 Arabidopsis chromosomes (chr1, chr2, chr3, chr4, chr5) and on Chloroplast and Mitochondria chromosome (chrC, chrM). As most of the original data is still in waiting period status, because of legal regulations it cannot be redistributed or repackaged. Thus we are not allowed to make the resultant FASTA sequences (or scripts to produce them) publicly available.

The information about 775 strains was gathered from four subprojects:

- 80 strains from "MPICao2010 - 80 Arabidopsis thaliana accessions", release 2012_03_13.

  Data access:
  `http://1001genomes.org/data/MPI/MPICao2010/releases/2012_03_13/strains/`

  Project's publication:
  Cao, J., Schneeberger, K., Ossowski, S., Gnther, T., Bender, S., Fitz, J.,

Koenig, D., Lanz, C., Stegle, O., Lippert, C., Wang, X., Ott, F., Mller, J., Alonso-Blanco, C., Borgwardt, K., Schmid, K. J., and Weigel, D. Whole-genome sequencing of multiple Arabidopsis thaliana populations. Nature Genetics 43, 956963 (2011).

Variants used:
– all 1-, 2- and 3-symbol insertions (accessible in `insertion.txt` file for each strain, no quality data available),
– filtered SNPs and 1-symbol deletions (accessible in `filtered_variant.txt` file for each strain, variants with quality greater or equal to 25 were processed).

- 170 z "Salk - Arabidopsis thaliana strains sequenced by the Salk Institute" (data about one strain is corrupted), release 2011_06_28.

  Data access:
  `http://1001genomes.org/data/Salk/releases/2011_06_28/TAIR10/strains/`

  This data was generated by the Salk Institute.

  Project's publication:
  Schmitz, R. J., Schultz, M. D., Urich, M. A., Nery, J. R., Pelizzola, M., Libiger, O., Alix, A., McCosh, R. B., Chen, H., Schork, N. J., and Ecker, J. R. (2013). Patterns of population epigenomic diversity. Nature 495, 193-198.

  Variants used:
  – all SNPs and 1-symbol deletions (accessible in `quality_variant_filtered_[ACCESSION].txt` file for each strain, all were with quality greater or equal to 25).

- 180 strains from "GMINordborg2010 - Arabidopsis thaliana strains sequenced by the Gregor Mendel Institute", release 2011_08_04.

  Data access:
  `http://1001genomes.org/data/GMI/GMINordborg2010/releases/2011_08_04/strains/`

  These sequence data were produced by the Nordborg laboratory at the Gregor Mendel Institute of Molecular Plant Biology.

  Project's publication:
  Long, Q., Rabanal, F. A., Meng, D., Huber, C. D., Farlow, A., Platzer, A., Zhang, Q., Vilhjalmsson, B. J., Korte, A., Nizhynska, V., Voronin, V., Korte, P., Sedman, L., Mandakova, T., Lysak, M. A., Seren, U., Hellmann, I., and Nordborg, M. (2013). Massive genomic variation and strong selection in Arabidopsis thaliana lines from Sweden. Nature Genetics, published online.

  Variants used:
  – all SNPs (accessible in `[ACCESSION].0cf.snp.log` file for each strain, no quality data available).

- 345 strains from "MPICWang2013 - 343 Arabidopsis thaliana accessions" (data about 345 more strains available), release 2013_04_15.

  Data access:
  `http://1001genomes.org/data/MPI/MPICWang2013/releases/2013_04_15/strains/`

These sequence data were produced by Monsanto Company and the Weigel laboratory at the Max Planck Institute for Developmental Biology.

Variants used:
– filtered SNPs and 1-symbol deletions (accessible in `quality_variant_[ACCESSION]_TAIR10.txt` file for each strain, variants with quality greater than or equal to 25 were processed).

1.3 The *H.sapiens alternate assemblies* dataset

The *H.sapiens alternate assemblies* dataset contains 11 different assemblies of the human genome. They are compressed against single reference sequence (other than other genomes in the set).

1.3.1 Reference sequence

The patch release 13 of Genome Reference Consortium Human Build 37 (GRCh37.p13, also referred to as hg19, released in 2013) was used as a reference sequence. It can be found at the GenBank and downloaded from the NCBI's anonymous FTP server:
`ftp://ftp.ncbi.nlm.nih.gov/genbank/genomes/Eukaryotes//vertebrates_mammals/Homo_sapiens/GRCh37.p13/Primary_Assembly/assembled_chromosomes/FASTA/`
The complete list of compressed reference sequences:
`chr1.fa.gz`, `chr2.fa.gz`, `chr3.fa.gz`, `chr4.fa.gz`, `chr5.fa.gz`, `chr6.fa.gz`, `chr7.fa.gz`, `chr8.fa.gz`, `chr9.fa.gz`, `chr10.fa.gz`, `chr11.fa.gz`, `chr12.fa.gz`, `chr13.fa.gz`, `chr14.fa.gz`, `chr15.fa.gz`, `chr16.fa.gz`, `chr17.fa.gz`, `chr18.fa.gz`, `chr19.fa.gz`, `chr20.fa.gz`, `chr21.fa.gz`, `chr22.fa.gz`, `chrX.fa.gz`, `chrY.fa.gz`.

1.3.2 Assembled genomes

The 11 FASTA sequences are different assemblies of the *H.sapiens* genome. The following genome assemblies were used:

- hg17 (also referred to as NCBI35.1)

  The past version of human genome reference sequence, NCBI Build 35, produced by the International Human Genome Sequencing Consortium (released in 2004). Data access:
  `ftp://ftp.ncbi.nih.gov/genomes//H_sapiens/ARCHIVE/BUILD.35.1/Assembled_chromosomes/*.fa.gz`

- hg18 (also referred to as NCBI36.3)

  The past version of human genome reference sequence assembly, NCBI Build 36.3, produced by the International Human Genome Sequencing Consortium (released in 2008). Data access:
  `ftp://ftp.ncbi.nih.gov/genomes//H_sapiens/ARCHIVE/BUILD.36.3/Assembled_chromosomes/*.fa.gz`

- hg38 (also referred to as GRCh38.p3)

  The latest version of the Human Reference by Genome Reference Consortium, Human Build 38.2 (released in 2015). Data access:
  `ftp://ftp.ncbi.nlm.nih.gov/genbank/genomes/Eukaryotes//vertebrates_mammals/Homo_sapiens/GRCh38.p2/Primary_Assembly/assembled_chromosomes/FASTA/*.fa.gz`

- CHM1_1.0

  The reference-guided de novo assembly based on human assembly GRCh37, produced by Washington University School of Medicine (released in 2012). Data access:

  `ftp://ftp.ncbi.nlm.nih.gov/genbank/genomes/Eukaryotes//vertebrates_mammals/`
  `/Homo_sapiens/CHM1_1.0/Primary_Assembly/assembled_chromosomes/FASTA/*.fa.gz`

- CHM1_1.1

  Reference-guided assembly based on human assembly GRCh37, produced by Washington University School of Medicine (released in 2013). Data access:

  `ftp://ftp.ncbi.nlm.nih.gov/genbank/genomes/Eukaryotes//vertebrates_mammals/`
  `Homo_sapiens/CHM1_1.1/Primary_Assembly/assembled_chromosomes/FASTA/*.fa.gz`

- HuRef

  The whole genome shotgun assembly by J. Craig Venter Institute. This assembly represents a composite haploid version of the diploid genome sequence from a single individual, J. Craig Venter (released in 2007). Data access:

  `ftp://ftp.ncbi.nih.gov/genomes//H_sapiens/ARCHIVE/ANNOTATION_RELEASE.106/`
  `Assembled_chromosomes/seq/hs_alt_HuRef*.fa.gz`

- WGSA

  The whole genome shotgun assembly of the human genome, produced by the Celera Genomics (released in 2001). Data access:

  `http://www.ncbi.nlm.nih.gov/assembly/GCA_000002115.2/`

- CSA

  The January 2001 compartmental shotgun assembly of the human genome (released in 2004). Data access:

  `http://www.ncbi.nlm.nih.gov/assembly/GCA_000365445.1/`

- YH

  The de novo assembled genome of an Asian individual, produced by Beijing Genomics Institute (released in 2010). Data access:

  `ftp://public.genomics.org.cn/BGI/yanhuang/fa/*fa.gz`

- KOREF_20090224

  The Korean individual genome sequence, KOREF_20090224 assembly (released in 2009). Data access:

  `ftp://ftp.kobic.kr/pub/KOBIC-KoreanGenome//KOREF_20090224/fasta/*fa.gz`

- KOREF_20090131

  The Korean individual genome sequence, KOREF_20090131 assembly (released in 2009). Data access:

  `ftp://ftp.kobic.kr/pub/KOBIC-KoreanGenome//KOREF_20090131/fasta/*.consensus_fasta`

2 GDC 2 package description

Genome Differential Compressor 2 (GDC 2), can be download in a `gdc2.tar.gz` package, which is is publicly available under a free license at `http://sun.aei.polsl.pl/REFRESH/index.php?page=projects&project=gdc&subpage=about`. It includes all programs and scripts presented in this section.

The following commands should be used to decompress the package and go to the main package directory:

```
tar -xzf gdc2.tar.gz
cd gdc2
```

The main package directory contains two folders:

1. `gdc_2`,
   containing main GDC 2 program (see Section 2.1),
2. `vcf2fasta`,
   containing scripts to retrieve FASTA sequences from Phase 1 of the 1000GP (see Section 2.2).

2.1 GDC 2

The GDC 2 program is in the `gdc_2` folder. It was implemented in C++11 language. Apart from proper compiler (e.g. gcc) and basic libraries, the compilation requires two specific libraries:

- asmlib, "A multi-platform library of highly optimized functions for C and C++"[1].
- zlib, "A Massively Spiffy Yet Delicately Unobtrusive Compression Library"[2]. The newest version (1.2.8) is required.

For linux and mac operating systems, the listed libraries are provided in the `libs` directory. However, the local versions may be used as well. The source codes and makefiles are placed in `Gdc2` folder. Two makefiles, `makefile.linux` and `makefile.mac`, can be used to build a linux or a mac os application, respectively. The source of the appropriate libraries can be changed there.

Instruction to build GDC 2 on linux:
```
make -f makefile.linux
```
Instruction to build GDC 2 on mac os:
```
make -f makefile.mac
```
Created `gdc2` executable is the GDC 2 program.

Basic usage:
```
gdc2 <mode> [options] <archive_name> [@list_of_files | file1_name file2_name ...]
```
where:

- `mode`
  chosen mode (required), `c` (compress), `d` (decompress) or `l` (list files in archive),

[1] http://www.agner.org/optimize/asmlib-instructions.pdf
[2] http://www.zlib.net

- `archive_name`
  archive name (required),
- `@list_of_files | file1_name file2_name ...`
  either name of file (`list_of_files`) containing a list of files (1 file per line) OR list of files separated by space (`file1_name file2_name ...`) to compress/decompress (required for `c` mode, optional for `d` mode),
- `options`:

  `-mpX,Y` – match parameters (default: 15,4), `X`—minimum length of 1st part of a match (hashing), `Y`—minimum length of 2nd and next parts of a match (after SNP/INS/DEL),

  `-i2` – additionally look for insertions/deletions (indels) of length 2 (default 1 only),

  `-tX` – set number of working threads to X (minimum is 2, default is 4),

  `-mmX` – set memory limit in MB that program can allocate to X (default is 1024MB),

  `-lX` – set compression degree (defining percentage of sequences used in the second level compression) to X, where X is an integer number in range [1-10] (default is 10); X×10 percent of sequences will be used in the second level compression.

Example usage:

```
gdc2 c my_archive @files_list.txt
gdc2 c -mp15,4 -i2 -t8 my_archive @files_list
gdc2 d my_archive @files_list
gdc2 d my_archive
gdc2 l my_archive
```

2.2 Scripts to retrieve FASTA sequences from the 1000GP

The `vcf2fasta` folder of the `gdc2` package contains scripts and tools to download Phase 1 1000GP data and generate FASTA sequences for all chromosomes of each individual in the set. The generated sequences are identical to the sequences used in the experiments described in the main paper. As explained in Section 1.1.2, because of the two pseudoautosomal regions (PAR1 and PAR2) that are shared between X and Y chromosomes in male individuals and method of storing information about variants in 1000GP VCF files, instead of processing chromosomes X and Y, we generate (and process) five groups of sequences, each treated as separate chromosome (X-fem, X-mal, Y-mal, XY-mal1, XY-mal2).

The minimal steps to retrieve FASTA sequences (for chromosomes specified by `CHROM` variable, which is set in `config.ini` file) in a unix environment with gcc compiler and basic utilities available, are:

```
cd src
make
cd ..
./run -abc
```

The whole processing requires about 9 TB of disk space (most of which is swallowed by 6.7 TB of consensus data and 1.2 TB of original VCF data). It should also be noted that the whole processing for all sequences may take a lot of time (several days).

It is possible to limit the processing to selected chromosomes in the configuration file `config.ini`. Possible chromosome names are: `1`, `2`, `3`, `4`, `5`, `6`, `7`, `8`, `9`, `10`, `11`, `12`, `13`, `14`, `15`, `16`, `17`, `18`, `19`, `20`, `21`, `22`, `X-fem`, `X-mal`, `X-mal1`, `X-mal2`, `Y-mal`. By default `$CHROM` variable is set to 22 (`CHROM="22"`), so only chromosome 22 will be handled. To process whole genome, all possible chromosome names must be listed by the `$CHROM` variable (`CHROM="1 2 3 4 5 6 7 8 9 10 11 12 13 14 15 16 17 18 19 20 21 22 X-fem X-mal X-mal1 X-mal2 Y-mal"`).

Two other variable in `config.ini`, `$FTP`, specifies the ftp site for download (EBI by default).

The available `run` switches:

- `-h`

  Presentation of possible switches of the `run` script. All options are briefly described.

- `-a`

  Download (`wget` utility) and decompression (`gzip` utility) of the reference sequences and VCF files. The VCF files are placed in `vcf` folder. References of chromosomes 1-22 are placed in the separate folder, dedicated to the specific chromosome (`chr1`, `chr2`, `chr3`, ...). References of chromosomes X and Y are placed in the main directory, as they require preprocessing (see option `-b`) before the main treatment. It should be noticed that the download of the whole genome requires about 1.2 TB of disk space.

- `-b`

  Preprocessing of the reference sequences and VCF files for X and Y chromosomes (which should be available in advance, see option `-a`). The reference FASTA sequences for PAR1, PAR2 and nonPAR regions are created with `cut-ref` (`chrX-mal.fa`, `chrXY-mal1.fa`, `chrXY-mal2.fa`, `chrY-mal.fa`). The `processX` program is used to create four VCF files, each describing variants corresponding to one of the X chromosome groups (X-fem, X-mal, X-mal1, X-mal2). The `cut` utility is used to remove data for the NA21313 (additional male individual, which does not occur in other VCF files) from the VCF file for Y chromosome.

- `-c`

  Creation of FASTA consensus sequences (extension `*.fa`) for all individuals from the reference sequence and VCF file (with use of the `VCF2FASTA-d` and/or `VCF2FASTA-h` programs). This is a very time consuming process and consensus sequences for all chromosomes require about 6.7 TB of disk space. The sequences are placed in the folders dedicated to the specific chromosome (`chr1`, `chr2`, `chr3`, ...).

Detailed description of all programs used by `run` script, together with their command line specifications:

- `cut-ref`

  The program creates a new FASTA file by cutting a piece of the input FASTA sequence and adding range information to the header. It is used to produce reference sequences for nonPAR, PAR1 and PAR2 regions of chromosomes X and Y, for male individuals. Usage:
  `cut-ref <input_name> <output_name> <start_pos> <end_pos>`
  `input_name` – name of the input file
  `output_name` – name of the output file
  `start_pos` – start position of the cut sequence
  `end_pos` – end position of the cut sequence

- `processX`

  The program processes the VCF file for X chromosome to create four VCF files: one for female individuals and three for male individuals. Each VCF file for male individuals corresponds to one of the region of interest of the chromosome X (PAR1, PAR2, nonPAR). Usage:
  `processX <vcf>`
  `vcf` – name of the VCF file for X chromosome

- `VCF2FASTA-h` and `VCF2FASTA-d`

  The programs process the input reference sequence of a chromosome and corresponding VCF file with haploid (`VCF2FASTA-h`) or diploid (`VCF2FASTA-d`) genotype calls to create one (`VCF2FASTA-h`) or two (`VCF2FASTA-d`) FASTA consensus sequence(s) for each individual described in the VCF file. Names of the output consensus sequences are made by adding to the name of the VCF file: individual's ID, chromosome indicator in case for autosomal chromosomes ('1' or '2', `VCF2FASTA-d` only) and the ".fa" extension. All variants are taken into account, regardless of the value of the FILTER field (only a warning is outputted if it's different than "PASS" or "."). Usage:
  `VCF2FASTA-h <vcf> <ref> [start_pos]`
  `VCF2FASTA-d <vcf> <ref> [start_pos]`
  `vcf` – name of the VCF file
  `ref` – name of the reference sequence
  `start_pos` – start position of the reference sequence (optional, 1 by default)

3 Parameters of programs used in the experiments

The parameters used for tools compressing FASTQ files:

- 7z a -md1024m — all EOL characters from input data were removed prior to compression
- RLZ files - l 1
- GReEn

- ABRC files ref_file 1 1
- GDC -ma1000000000 -rn1 — denoted as GDC-normal
- GDC -ma1000000000 -rn40 — denoted as GDC-ultra
- FRESCO ./config.ini COMPRESS ./ ./FR.out/
  FRESCO ./config.ini SOCOMPRESS ./FR.out ./FR.so/
  The number of additional references was set to 100
- iDoComp — Generation of the suffix array for the reference sequence:
  ./generateSA.sh ./sa/ ./sa
  Compression of the collection of FASTA files:
  ./iDoComp.run c iDoComp.txt /fasta
- gdc2 c archive @filex.txt

4 Additional figures

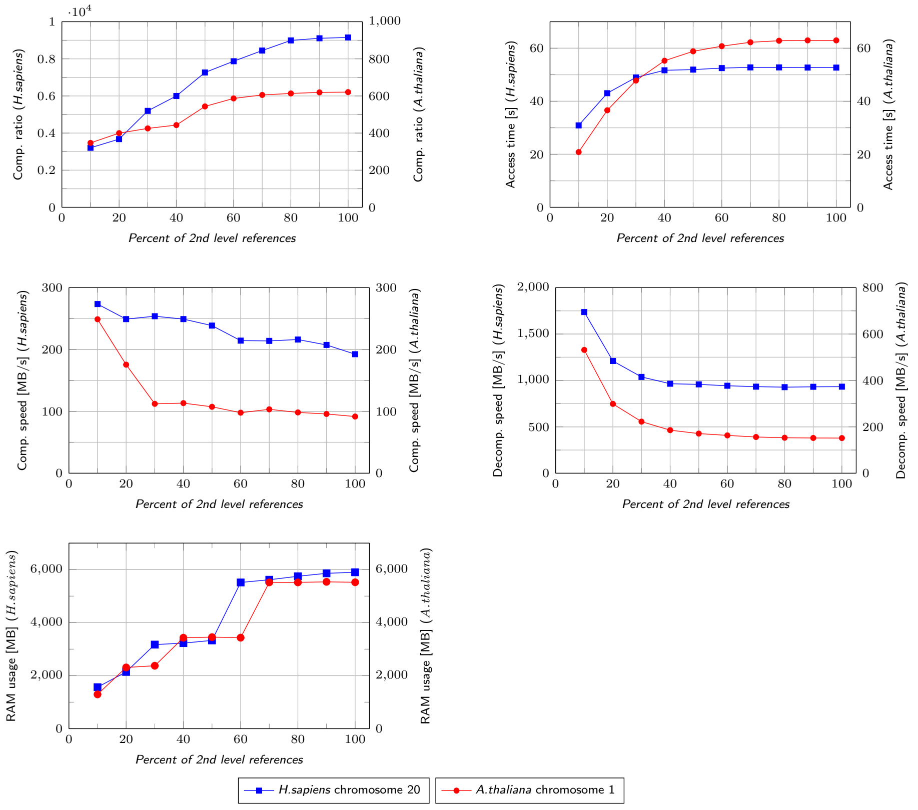

Supplementary Figure S1: Influence of the percent of 2nd level references on compression ratio, decompression (access) time of a single sequence, compression speed, decompression speed and RAM usage.
